# The BDNF-TrkB Pathway Acts Through Nucleus Accumbens D2 Expressing Neurons to Mediate Stress Susceptible Outcomes

**DOI:** 10.3389/fpsyt.2022.854494

**Published:** 2022-06-02

**Authors:** Marco Pagliusi, Daniela Franco, Shannon Cole, Gessynger Morais-Silva, Ramesh Chandra, Megan E. Fox, Sergio D. Iñiguez, Cesar R. Sartori, Mary Kay Lobo

**Affiliations:** ^1^Department of Anatomy and Neurobiology, University of Maryland School of Medicine, Baltimore, MD, United States; ^2^Department of Structural and Functional Biology, University of Campinas, Campinas, Brazil; ^3^School of Pharmaceutical Sciences, São Paulo State University, Araraquara, Brazil; ^4^Department of Psychology, University of Texas at El Paso, El Paso, TX, United States

**Keywords:** medium spiny neurons, social defeat stress, witness defeat, depression, resilience, nucleus accumbens (NAc)

## Abstract

Brain-derived neurotrophic factor (BDNF) has a critical role in stress response including neuropsychiatric disorders that are precipitated by stress, such as major depressive disorder (MDD). BDNF acts through its full-length BDNF receptor tyrosine kinase B (TrkB) to trigger a pro-plasticity effect. In contrast, the truncated isoform of the BDNF receptor (TrkB.t1) triggers an anti-plasticity effect. In stress outcomes, BDNF acting in the hippocampus has a stress resilience effect, and, inversely, in the nucleus accumbens (NAc), BDNF acts as a stress susceptible molecule. It is unknown if BDNF-TrkB acts on a specific NAc projection neuron, i.e., medium spiny neuron (MSN or spiny projection neuron), a subtype in stress outcomes. To determine this, we performed chronic social or vicarious witness defeat stress (CSDS or CWDS) in mice expressing TrkB.t1 in dopamine receptor 1 or 2 containing MSNs (D1- or D2-MSNs). Our results showed that TrkB.t1 overexpression in NAc D2-MSNs prevented the CSDS-induced social avoidance or other stress susceptible behaviors in male and female mice. We further showed that this overexpression in D2-MSNs blocked stress susceptible behavior induced by intra-NAc BDNF infusion. In contrast, our results demonstrate that overexpression of TrkB.t1 on NAc D1-MSNs facilitates the SDS susceptible behaviors. Our study provides enhanced details into the NAc cell subtype role of BDNF-TrkB signaling in stress outcomes.

## Introduction

Brain-derived neurotrophic factor (BDNF) is an important pro-plasticity and cellular survival neurotrophic factor that has been implicated in several brain disorders (1–3). In major depressive disorder and related stress models, BDNF is associated with pro- and antidepressive-like, as well as stress susceptible and resilient, outcomes, depending on the brain region it is acting (4–7). More importantly, BDNF acting in the hippocampus reduces negative affective states and stress susceptibility, and BDNF acting in the dopaminergic mesolimbic pathway [e.g., nucleus accumbens (NAc)] leads to enhanced stress susceptible outcomes (4–8). Chronic social defeat stress (CSDS) increases BDNF levels in the NAc of susceptible mice, which displays social avoidance and anhedonia (7, 8). Furthermore, intra-NAc BDNF infusion increases susceptibility to social stress, inducing social avoidance after a subthreshold social defeat stress (SSDS) paradigm (6). In contrast, knockdown or reduction of VTA-NAc BDNF levels prevents CSDS stress susceptible outcomes (6, 7).

The pro-plasticity effects of BDNF are triggered by the activation of the full-length BDNF receptor tyrosine kinase B (here called TrkB), initiating a specific intracellular signaling pathway (6, 9, 10). *TrkB* is expressed from one gene and is available in the cells as full length (TrkB) and truncated isoforms, such as TrkB.t1 (also known as glycoprotein 95), TrkB.t2, TrkB.t3, and TrkB.t4 (10–12). The role of TrkB.t2, TrkB.t3, and TrkB.t4 in BDNF signaling is unknown, and it is unclear if these isoforms are translated into functional proteins (10). However, TrkB.t1 acts as an anti-plasticity receptor (1) since it lacks the tyrosine kinase-signaling domain and cannot trigger the TrkB intracellular signaling cascade (1). TrkB.t1 can inhibit TrkB signaling, mainly by sequestering BDNF, with a consequential decrease in downstream signaling pathway proteins (e.g., PLCg, PI3K, and Erk/MAPK) (1, 10). This TrkB.t1 dominant-negative inhibition of TrkB-signaling is so well-characterized that many studies use TrkB.t1 overexpression as an approach to assess TrkB-related phenomena (1). For example, this approach was used to first demonstrate that BDNF acting in the dopaminergic mesolimbic system, specifically in VTA-NAc projections, triggers a stress susceptible outcome (13).

Although it is known that BDNF in the NAc triggers a pro-susceptibility outcome, it is unclear in which medium spiny neuron (MSN) subtype BDNF acts to promote this effect. The striatum (which includes NAc) is mainly composed of MSNs, which are distinguished by their differential enrichment of many molecules including the expression of dopamine receptor D1 (D1-MSN) or D2 (D2-MSN) (14–16). These MSN subtypes are also distinguished by their projections, where D1-MSNs project to ventral tegmental area (VTA), ventral pallidum (VP), and lateral hypothalamus (LH), while D2-MSNs project to VP and LH (17–19). Given this lack of knowledge into BDNF-TrkB actions in NAc MSN subtypes in stress, we expressed TrkB.t1 in MSN subtypes during SDS to examine the impact of blocking BDNF-TrkB signaling in these neurons on stress outcomes.

## Materials and Methods

### Experimental Subjects

Male or female Drd1a-Cre (D1-Cre, Line FK150) or Adora2a-Cre (A2A-Cre, Line KG139) hemizygote bacterial artificial chromosome transgenic mice on a C57BL/6J background were 8 weeks old in the beginning of each behavioral experiment. CD1 retired breeders (Charles River, Raleigh, NC, United States) were used as residents in the social defeat stress protocol and were obtained at ∼3 months old (they were ≥ 3 months old at the start of the experiment). Mice were maintained on a 12-h light/dark cycle with *ad libitum* food and water. Housing and behavioral conditions occurred in a room with < 40 dB. All studies were approved and conducted in accordance with the guidelines set up by the Institutional Animal Care and Use Committee at the University of Maryland School of Medicine.

### Stereotaxic Surgery

Experimental mice were anesthetized with isoflurane (3–5% induction and 1–3% maintenance) using a mouse anesthesia machine to inject serotype 9 adeno-associated viruses (AAV) TrkB.t1. ORF was PCR amplified using Phusion DNA polymerase (New England Biolabs) and cloned into the *Nhe*I and *Nco*I restriction site of the destination AAV-EF1a-DIO-EYFP vector. Finally, AAV-DIO-Ef1a-TrkB.t1-eYFP and AAV9-DIO-Ef1a-eYFP were packaged into AAV (serotype 9) at UMB Virus Vector Core Facility. D1-Cre and A2A-Cre mice received bilateral stereotaxic injections into the NAc (anterior/posterior: +1.6, lateral: +1.5, dorsal/ventral: −4.4, angle: 10°) with a Cre-inducible (double inverted open reading frame, DIO) AAV to express truncated TrkB (TrkB.t1) isoform (AAV9-DIO-Ef1a-TrkB.t1-eYFP, titer 4.1E + 12 GC/ml) or control AAV to express only the reporter gene (AAV9-DIO-Ef1a-eYFP, titer 3 × 10*12 GC/ml). Virus was infused at a rate of 0.1 μl/min in a total volume of 0.5 μl. The injection needle was left in place for 8 min following the infusion. For experiments requiring cannula implantation, mice were implanted with a 26-gauge bilateral guide cannula following virus injection.

### Chronic Social Defeat Stress

Social defeat stress is a well-established, ethologically relevant stress protocol to induce social avoidance in male mice (7, 20). Briefly, our experimental mice were exposed daily to an aggressive CD1 mouse for 10 min over 10 consecutive days, each day exposed to a different resident CD1 mouse. After this period of physical contact, both stressed C57BL/6J mouse and resident CD1 mouse remained for 24 h in the same cage but were separated by a clear perforated divider allowing only sensorial contact. Control non-stressed mice were handled throughout the 10-day protocol period similarly to the stressed groups with no social contact and paired with another C57BL/6J mouse.

### Subthreshold Social Defeat Stress

Subthreshold social defeat stress is widely used to investigate stress susceptibility after a molecular manipulation (6, 21, 22). In this protocol, our experimental mice were exposed to an aggressive CD1 mouse for three sessions in a row of 5 min of physical contact + 15 min of sensorial contact, each session facing a different resident CD1 mouse. This 1-day SDS protocol does not induce depressive-like behavior of social avoidance in naïve mice.

### Chronic Witness Defeat Stress

Chronic witness or vicarious defeat stress was previously standardized and shown to induce stress susceptible behavior in male and female mice (23–25). Briefly, female C57BL/6J mice witness the social defeat of a conspecific male mouse by an aggressive CD1 male for 10 consecutive days (but only with sensorial contact through a transparent perforated acrylic divider) (26). All social defeat and witness sessions last for 10 min, and following the session, the female mice remained in the cage with sensory contact with the aggressor CD1.

### Social Interaction Test

Social interaction (SI) test was performed as previously described after CSDS or SSDS (20). Briefly, this test was performed in two 2.5-min sessions using an open field arena (42 × 42 × 42 cm) containing an interaction zone with a perforated plastic enclosure and two corner zones. The first *no-target* session examines time in the interaction zone, where an empty perforated plastic enclosure is located. The second *target* session examines time in the interaction zone, where a perforated plastic enclosure with an unknown CD1 social target is located. Both sessions were recorded by TopScan video tracking software (CleverSys, Reston, VA, United States) to assess the total time spent in the interaction and corner zones.

### Three-Chamber Social Test

The three-chamber social test was performed to investigate social behavior in female mice. This test was performed as previously described (27). Briefly, this test is performed using a three-chamber arena; each chamber is sized 30.5 × 19.5 × 31.0 cm and is made with clear acrylic Plexiglas. First, the experimental mouse was placed in the center chamber and allowed free exploration in the entire arena (all three chambers) for 5 min. After this first session, our experimental mouse was removed from the arena for 30 s, and an unfamiliar female mouse was placed in one of the side chambers (social chamber) inside a perforated plastic enclosure, while an empty plastic enclosure remained in the opposite side chamber (counterbalanced across experimental mice). Subsequently, the same experimental female mouse was placed in the center chamber for the second session and was allowed for free exploration in the entire arena. Both sessions were recorded by TopScan video tracking software (CleverSys, Reston, VA, United States) to assess the total time spent in the interaction, center, and empty chambers.

### Sucrose Splash Test

This test is pharmacologically validated to investigate motivational behavior and was performed as previously described (28). Briefly, in this test we sprayed a 10% sucrose solution on the dorsal coat of the experimental mice and scored total time spent grooming for 5 min.

### Sucrose Preference

The sucrose preference test is a well-established test to investigate anhedonia. We used the two-bottle choice preference paradigm as previously described (21). Briefly, our experimental mice were first habituated to two bottles of water for 24 h. Following this habituation period, one bottle was filled with 1% sucrose solution, and on the next day, the bottles were alternated to opposite sides. Both bottles were weighed every day to assess the preference (%) for sucrose solution. All results are shown as the preference for sucrose solution on the last day of the test.

### Forced Swim Test

In this test, our experimental mice were individually placed in a transparent glass cylinder (5 L beaker) filled with 3 L of filtered water (23–25°C). The session was recorded for 6 min, and the total time spent immobile was determined.

### Immunofluorescence

Immunofluorescence was performed to verify the virus injection sites. Our experimental mice were perfused with 1 × phosphate-buffered saline (PBS) followed by 4% paraformaldehyde diluted in 1 × PBS. Brains were extracted and left in paraformaldehyde solution overnight. Next, all brains were stocked in 0.01% sodium azide diluted in 1 × PBS solution. All brains were sliced in 40 μm using a vibratome (Leica, Buffalo Grove, IL, United States), and immunofluorescence was performed as previously described (21, 29). Briefly, the slices were blocked for 30 min in 0.3% Triton and 3% normal donkey serum and then incubated overnight (4°C) in 1:1,500 chicken anti-GFP primary antibody (CellSignaling, Danvers, MA, United States). The slices were rinsed with 1 × PBS and incubated for 2 h in secondary antibody: 1:1,000 anti-chicken Alexa488 (Invitrogen, Carlsbad, CA, United States). Immunofluorescence imaging was performed on an Olympus Bx61 confocal microscope.

### Statistical Analyses

Analyses were performed using the Graphpad Prism 6 software (La Jolla, California). Behavioral data were analyzed by unpaired *t*-test, two-way ANOVA, or repeated measures ANOVA followed by *post-hoc* Tukey’s multiple comparisons test. Data are presented as mean ± standard error of mean (SEM), and the statistically significant difference was considered when *p* ≤ 0.05.

## Results

### Blockade of BDNF-TrkB Signaling in D2-MSNs Causes Resilient Outcomes to Social Stress in Both Sexes

**FIGURE 1:**
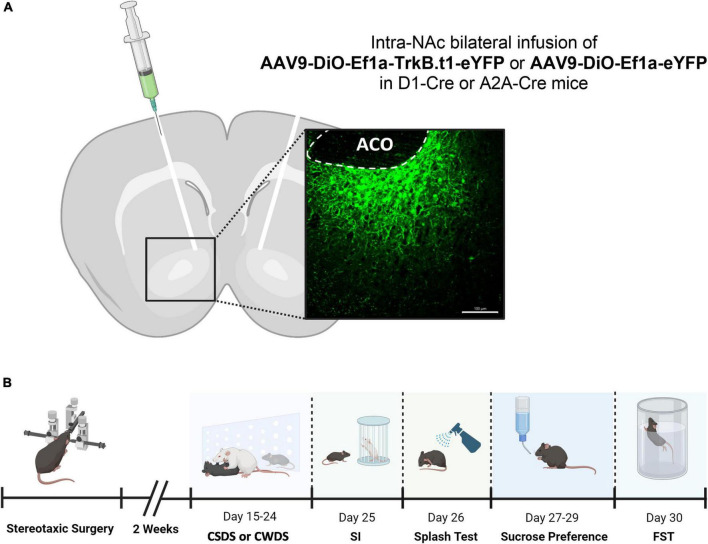
Injection sites for intra-NAc virus vector infusion and behavioral experimental design. **(A)** Viral vectors and image demonstrating the viral injection, with EYFP expression virus, scale bar = 100 μm (aco, anterior commissure). **(B)** Behavioral experimental design showing the sequence of the tests performed in this experiment (SI, social interaction test; FST, forced swim test).

**FIGURE 2:**
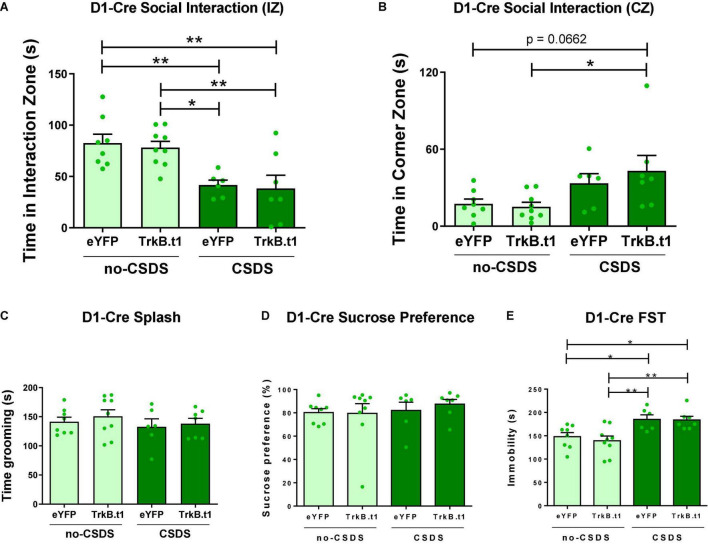
Blocking BDNF-TrkB signaling in D1-MSNs did not induce a resilient phenotype after 10 days of social defeat stress (CSDS); instead, it induced a susceptible phenotype. **(A)** Both CSDS groups spent less time interacting with the social target, showing social avoidance (*N* = 6–9). **(B)** CSDS-D1-MSN-TrkB.t1 group spent more time in the corner during the presence of the social target when compared with no-CSDS (*N* = 6–9). **(C,D)** Total time spent grooming during the sucrose splash test or sucrose preference was not significantly different across groups (*N* = 6–9). **(E)** Both CSDS groups demonstrated more time immobile in the forced swim test when compared with no-CSDS groups (*N* = 6–9) (*p ≤ 0.05; **p ≤ 0.01).

D1-Cre and A2A-Cre (the latter expressing Cre in D2-MSNs but not D2 cholinergic interneurons) male and female mice received an infusion of AAV-DIO-Trkb.t1-eYFP or AAV-DIO-Trkb.t1-eYFP into the NAc (Figure 1A) followed by 10 days of social or witness defeat stress and downstream behavioral analysis (Figure 1B). While both CSDS D1-Cre mice displayed reduced time in the interaction zone with a novel social target, there was no effect of Trkb.t1 expression in D1-MSNs on social interaction (Figure 2A). Two-way ANOVA showed a significant difference in the stress factor [*F*_(1,26)_ = 21.45; *p* ≤ 0.0001], and the *post-hoc* Tukey’s multiple comparisons test revealed statistical differences between CSDS-eYFP and both no-CSDS groups (*p* ≤ 0.05 vs. TrkB.t1 and *p* ≤ 0.01 vs. eYFP), and also between CSDS-TrkB.t1 and both no-CSDS groups (*p* ≤ 0.01), showing that both CSDS groups spent less time interacting with the social target. Also, mice with TrkB.t1 expression in D1-MSNs were significantly different in time in the corner zone during the target session when compared with the no-CSDS group (Figure 2B). Two-way ANOVA showed a significant difference considering the stress factor [*F*_(1,26)_ = 9.79; *p* = 0.004], and the *post-hoc* Tukey’s multiple comparisons test revealed statistical differences between CSDS-TrkB.t1 and no-CSDS-TrkB.t1 (*p* ≤ 0.05), showing that CSDS-TrkB.t1 spent more time avoiding the social interaction with the social target. The total time in grooming during the sucrose splash test or sucrose preference was not altered between stress and virus groups (Figures 2C,D). However, immobility time was significantly increased during the forced swim test in both CSDS groups, and therefore, no effect of TrkB.t1 expression in D1-MSNs was observed (Figure 2E). Two-way ANOVA revealed significant differences considering the stress factor [*F*_(1,26)_ = 18.39; *p* = 0.002], and the *post-hoc* Tukey’s multiple comparisons test revealed statistical differences between no-CSDS-eYFP and both CSDS groups (*p* ≤ 0.05) and also between no-CSDS-TrkB.t1 and both CSDS groups (*p* ≤ 0.01), showing that CSDS groups spent more time in immobility during this test.

**FIGURE 3:**
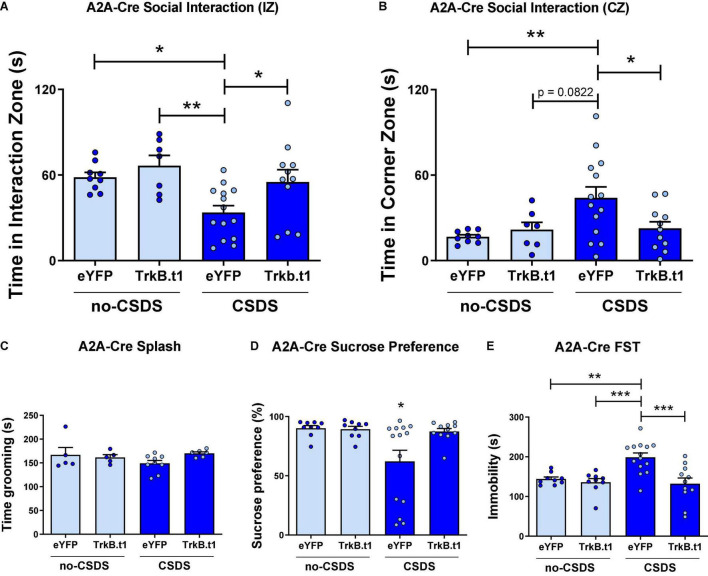
Blocking BDNF-TrkB signaling in D2-MSNs induces a resilient phenotype after 10 days of chronic social defeat stress (CSDS) in male mice. **(A)** CSDS-eYFP group spent less time interacting with the social target (target session), a behavior prevented by TrkB.t1 expression in D2-MSNs (*N* = 7–14). **(B)** CSDS-eYFP group spent more time avoiding social interaction with the social target (target session), which was also prevented by TrkB.t1 expression in D2-MSNs (*N* = 7–14). **(C)** Total time spent in grooming during the sucrose splash test was not significant across groups (*N* = 5–7). **(D,E)** TrkB.t1 expression in D2-MSNs prevented the CSDS-induced anhedonia and despair behavior (*N* = 9–14) (*p ≤ 0.05; **p ≤ 0.01; ***p ≤ 0.001).

In contrast to D1-Cre-Trkb.t1 conditions, overexpression of Trkb.t1 in D2-MSNs using A2A-Cre mice prevented the social interaction impairment after CSDS (Figure 3A). Two-way ANOVA showed significant differences considering stress factor [*F*_(1,37)_ = 7.602; *p* = 0.009] and virus factor [*F*_(1,37)_ = 5.045; *p* = 0.031], and the *post-hoc* Tukey’s multiple comparisons test revealed statistical differences between CSDS-eYFP and other groups (*p* ≤ 0.05 vs. no-CSDS-eYFP and CSDS-TrkB.t1; *p* ≤ 0.01 vs. no-CSDS-TrkB.t1), showing that CSDS-eYFP mice spent less time interacting with the social target. TrkB.t1 expression in D2-MSNs also prevented the CSDS-induced increase in total time spent in the corner zones during the target session of the social interaction test (Figure 3B). Two-way ANOVA showed significant differences considering stress factor [*F*_(1,37)_ = 4.988; *p* = 0.032], and the *post-hoc* Tukey’s multiple comparisons test revealed statistical differences between CSDS-eYFP and other groups (*p* ≤ 0.05) with the exception of no-CSDS-TrkB.t1 (*p* = 0.082), showing that CSDS-eYFP spent more time avoiding the social interaction with the social target. The total time in grooming during the sucrose splash test was not altered between stress and virus groups (Figure 3C). TrkB.t1 expression in D2-MSNs prevented the CSDS-induced anhedonia and despair behavior, respectively, assessed in the sucrose preference test and forced swim test (Figures 3D,E). Two-way ANOVA showed significant differences considering stress factor [*F*_(1,37)_ = 5.164; *p* = 0.029], and the *post-hoc* Tukey’s multiple comparisons test revealed statistical differences between CSDS-eYFP and other groups (*p* ≤ 0.05), showing less preference to sucrose 1% solution for this group. Two-way ANOVA showed significant differences in stress [*F*_(1,39)_ = 5.183; *p* = 0.028] and virus factor [*F*_(1,39)_ = 11.12; *p* = 0.0019], and the *post-hoc* Tukey’s multiple comparisons test revealed statistical differences between CSDS-eYFP and other groups (*p* ≤ 0.01 vs. no-CSDS-eYFP; *p* ≤ 0.001 vs. no-CSDS-TrkB.t1 and CSDS-TrkB.t1), showing that CSDS-eYFP spent more time in immobility during this test.

**FIGURE 4:**
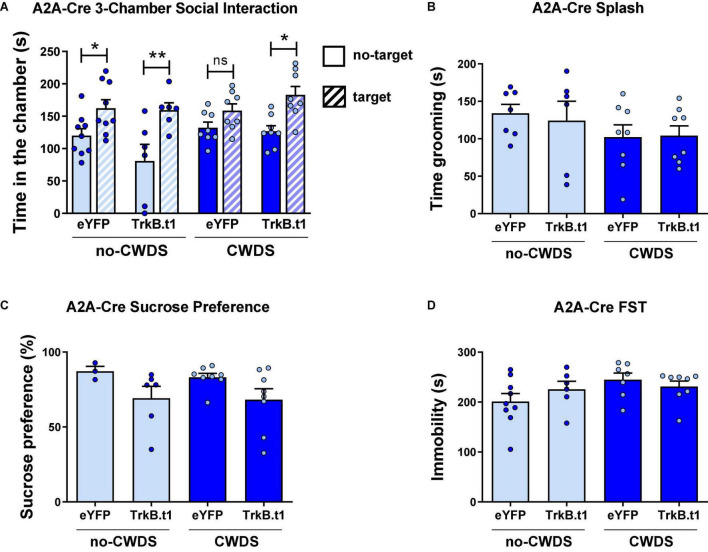
Blocking BDNF-TrkB signaling in D2-MSNs induces a resilient phenotype after 10 days of chronic witness defeat stress (CWDS) in female mice. **(A)** CWDS-eYFP group did not show preference for the novel social target during the 3-chamber social test, a behavior prevented by TrkB.t1 expression in D2-MSNs (*N* = 6–9). **(B–D)** Total time in grooming during the sucrose splash test (*N* = 6–8), the sucrose preference test (*N* = 3–8), or the forced swim test (*N* = 6–9) was not significant across groups (*p ≤ 0.05; **p ≤ 0.01).

Female mice also showed a resilient phenotype following CWDS after TrkB.t1 expression in D2-MSNs (Figure 4A), in social behavior assessed by the three-chamber social test. Figure 4A shows that all groups except for eYFP-CWDS display preference for the social chamber when comparing the total time spent in this chamber during the no-target vs. target session. Repeated measures two-way ANOVA showed significant differences considering session (no-target vs. target) factor [*F*_(1,27)_ = 27.99; *p* ≤ 0.001] and virus factor [*F*_(3,27)_ = 3.51; *p* = 0.029]. Sidak’s multiple comparisons test revealed statistical differences between no-target and target sessions for all groups (*p* ≤ 0.05) except for eYFP-CWDS. When comparing the total time spent in the empty chamber during the no-target vs. target sessions, only TrkB.t1-no-CWDS group showed a reduction in the total time in the empty chamber (*p* ≤ 0.01) when the social target was present (see Supplementary Figure 2). The total time in grooming during the sucrose splash test, the sucrose preference test, or the forced swim test was not altered between stress and virus groups (Figures 4B–D).

### Blockade of BDNF-TrkB Signaling in D1-MSNs Induces a Susceptible Outcome After a Subthreshold Social Defeat Stress

**FIGURE 5:**
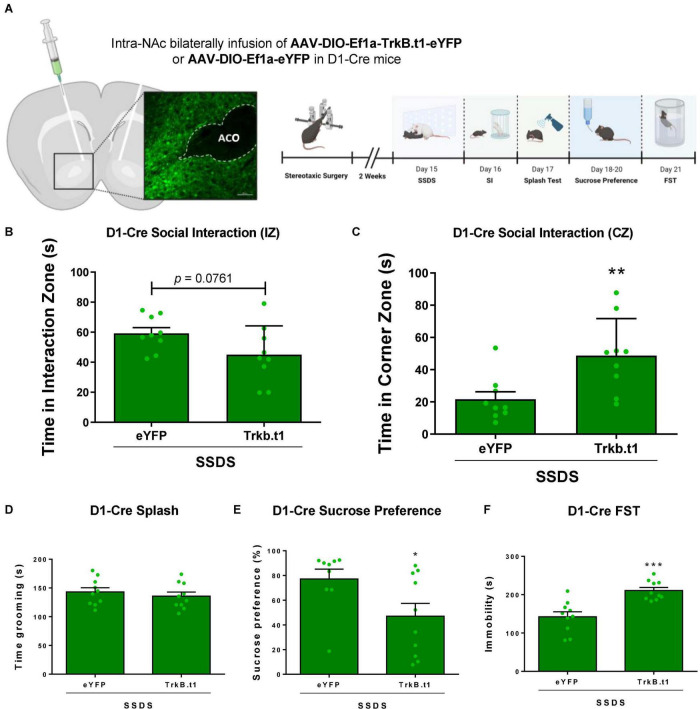
Blocking BDNF-TrkB signaling in D1-MSNs induces a susceptible phenotype after 1 day (subthreshold) of social defeat stress (SSDS) in male mice. **(A)** Viral vectors and image demonstrating the viral injection, with EYFP expression virus, scale bar = 100 μm. **(B,C)** SSDS-TrkB.t1 mice spent less time in the interaction zone and more time in the corner zone during the target session of the social interaction test after SSDS (*N* = 9 per group). **(D)** Total time grooming during the sucrose splash test was not significantly different across groups (*N* = 10 per group). **(E)** SSDS-TrkB.t1 mice showed less preference for the sucrose solution when compared with SSDS-eYFP (*N* = 9–10). **(F)** SSDS-TrkB.t1 mice showed more time immobile when compared with SSDS-eYFP (*N* = 9–10) (*p ≤ 0.05; **p ≤ 0.01; ***p ≤ 0.001; SSDS, subthreshold social defeat stress; SI, social interaction test; Splash, sucrose splash test; FST, forced swim test).

D1-Cre male mice received an infusion of AAV9-DIO-Trkb.t1-eYFP or AAV9-DIO-Trkb.t1-eYFP into the NAc followed by 1 day of social defeat stress (SSDS) and downstream behavioral analysis (Figure 5A). SSDS-TrkB.t1 group displayed social avoidance after SSDS (Figures 5B,C). While not significantly different in the time in the social interaction zone (*p* = 0.0761, *t* = 1.896, df = 16), a significant difference in time spent in the corner zone (*p* ≤ 0.01, *t* = 2.707, df = 16) during the target session of the social interaction test was observed. The total time grooming during the sucrose splash test was not altered between groups (Figure 5D). TrkB.t1 expression in D1-MSNs also predisposed mice for anhedonia and increased time spent immobile in the forced swim test following SSDS (Figures 5E,F). SSDS-TrkB.t1 group showed less preference to sucrose 1% solution (*p* ≤ 0.05, *t* = 2.263, df = 17) compared with SSDS-eYFP group. Finally, SSDS-TrkB.t1 group spent more time in immobile (*p* ≤ 0.001, *t* = 4.529, df = 18) compared with SSDS-eYFP group. We also compared the D1-Cre no-CSDS, a group that did not undergo any stress, and CSDS groups in Figure 2 with the SSDS groups in the current experiment (Supplementary Figure 1). The analysis demonstrates that both TrkB.t1 stressed groups (SSDS and CSDS) and eYFP-CSDS display social avoidance (*p* ≤ 0.01) when compared with no-CSDS (no stress) groups. In contrast eYFP-SSDS D1-Cre mice do not display social avoidance when compared to the no stress groups.

### Blockade of BDNF-TrkB Signaling in D2-MSNs Prevents the Intra-NAc BDNF-Induced Susceptibility

**FIGURE 6:**
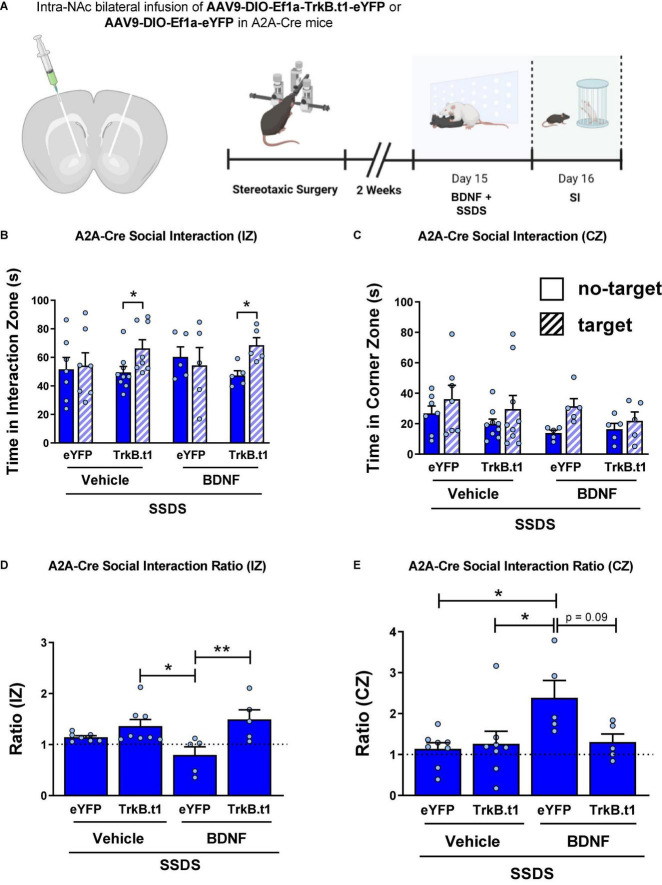
Blocking BDNF-TrkB signaling in D2-MSNs prevents the intra-NAc BDNF infusion-induced stress susceptibility in male mice. **(A)** Illustration of the viral injection site, the viral vectors used in this experiment (with cannula implantation), and the experimental design. **(B)** TrkB.t1 expression in D2-MSNs increased social interaction even after BDNF infusion and SSDS. **(C)** TrkB.t1 expression in D2-MSNs blocked BDNF-induced increase in corner zone preference after SSDS during the social interaction test. **(D)** Social interaction ratio in the interaction zone and in the **(E)** corner zone, showing that only SSDS-BDNF-eYFP mice showed a susceptible phenotype as previously described (*N* = 5–9 per group; *p ≤ 0.05; **p ≤ 0.01; SI, social interaction test; SSDS, subthreshold social defeat stress).

A2A-Cre male mice received an infusion of AAV9-DIO-Trkb.t1-eYFP or AAV9-DIO-Trkb.t1-eYFP into the NAc followed by 1 day of social defeat stress (SSDS) along with intra-NAc BDNF infusion followed by downstream behavioral analysis (Figure 6A). Our results demonstrate that intra-NAc BDNF infusion induced a susceptible-like phenotype in the eYFP group after SSDS, and TrkB.t1 overexpression in D2-MSNs blocked the BDNF effect. Figure 6B shows that both TrkB.t1 groups (vehicle and BDNF) spent more time in the interaction zone during the target session when compared with no-target session (*p* ≤ 0.05). Repeated measures two-way ANOVA showed significant differences considering session (no-target vs. target) factor [*F*_(1,21)_ = 6.61; *p* = 0.018] and subject [*F*_(21,21)_ = 2.95; *p* = 0.008]. Sidak’s multiple comparisons test revealed statistical differences between no-target and target sessions for both TrkB.t1 groups (*p* ≤ 0.05). Figure 6C shows that there is no significant difference between no-target and target sessions when considering the total time spent in the corners zone.

Figure 6D shows the social interaction ratio in the interaction zone, calculated by dividing the time spent in the interaction zone in the target session by the time spent in the interaction zone in the no-target session during the social interaction test. Two-way ANOVA showed a significant difference considering the virus factor [*F*_(1,21)_ = 12.07; *p* = 0.002], and the *post-hoc* Tukey’s multiple comparisons test revealed statistical differences between SSDS-BDNF-eYFP and both TrkB.t1 groups, SSDS-BDNF-TrkB.t1 (*p* ≤ 0.01) and SSDS-Veh-TrkB.t1 (*p* ≤ 0.05). We can see that only SSDS-BDNF-eYFP group showed social interaction ratio ≤ 1, a susceptible phenotype. Figure 6E shows the social interaction ratio in the corner zone, calculated by dividing the time spent in the corner zone in the target session by the time spent in the interaction zone in the no-target session during the social interaction test. Two-way ANOVA showed a significant difference in the BDNF injection factor [*F*_(1,21)_ = 5.23; *p* = 0.032], and the *post-hoc* Tukey’s multiple comparisons test revealed statistical differences between SSDS-BDNF-eYFP and other groups (*p* ≤ 0.05) except SSDS-BDNF-TrkB.t1 (*p* = 0.0996), showing that only SSDS-BDNF-eYFP group showed susceptible phenotype.

## Discussion

Our results demonstrate that truncated TrkB (TrkB.t1) BDNF receptor overexpression in NAc D2-MSNs prevents chronic social stress outcomes in male and female mice. We also showed that this overexpression blocked susceptibility induced by intra-NAc BDNF infusion. In contrast, our results demonstrate that overexpression of TrkB.t1 in NAc D1-MSNs facilitates stress susceptible outcomes after an SSDS paradigm. In this study, we used three different stress paradigms, namely, CSDS, CWDS, and SSDS. The first one is a standardized procedure ethologically relevant and is widely used to study stress outcomes in male mice (6, 7, 20, 30–32). The second is a standardized procedure used to study stress outcomes in female mice, which chronically witness a co-specific male mouse undergoing social defeat (23–25). SSDS is used to study stress susceptibility, using a 1-day social defeat stress that is not sufficient to induce susceptible outcomes in naïve mice (6, 21, 22, 33).

The central role of BDNF in depressive disorders and stress outcomes is well-established in the literature; however, BDNF exerts dynamic outcomes depending on the brain region or in this case cell type examined. BDNF signaling may have opposite effects on stress outcomes; enhanced BDNF signaling in hippocampus results in a stress resilience effect, and in dopaminergic mesolimbic structures (e.g., NAc), it causes a stress susceptible outcome (6, 7, 34, 35). Eisch and colleagues, for example, used TrkB.t1 overexpression to demonstrate that blocking BDNF-TrkB signaling in the NAc induced an antidepressive outcome in the forced swim test (13). Krishnan et al. (6) showed that CSDS-susceptible mice and humans with depression (*post mortem*) have more BDNF in the NAc when compared with control or no-depressive subjects. Furthermore, they showed an increase in the expression of BDNF-TrkB signaling cascade proteins, such as Akt, Gsk-3β, and ERK in the NAc of CSDS-susceptible mice and depressive humans (6). Previous work from this group by Berton et al. (7) showed that BDNF released from VTA projections to NAc is responsible for the susceptible phenotype after CSDS. Finally, blockade of BDNF-TrkB signaling in NAc blocks the social defeat stress susceptible outcome that occurs with phasic stimulation of VTA neurons projecting to NAc (36).

Despite the numerous studies that investigate the role of BDNF on the dopaminergic mesolimbic pathway in depressive disorder and stress outcome, this is the first study to investigate the role of BDNF-TrkB signaling in specific NAc-MSN subtypes. To do this, we suppressed the BDNF-TrkB signaling pathway using a Cre-dependent virus vector that overexpresses the truncated TrkB (TrkB.t1) isoform. This approach has been widely used to study BDNF-TrkB-related phenomena since TrkB.t1 exerts a dominant-negative inhibition on the TrkB signaling pathway (1).

NAc MSNs subtypes have overlapping but distinct projections and are composed of different molecular signatures, and through these characteristics, they can exert distinct influences on behavior (17, 21, 37, 38). For example, our group, using an optogenetic approach, showed that repeated high-frequency activation of D1-MSNs, and not D2-MSNs, in the NAc reverses social avoidance induced by CSDS (21). Furthermore, we also showed that resilient mice become susceptible after repeated inhibition of NAc D1-MSNs (21). In contrast, repeated and high-frequency stimulation of NAc D2-MSNs prior to an SSDS induces a susceptible phenotype, and susceptible mice display enhanced excitatory input and dendritic spine plasticity in D2-MSNs (21, 39). Additionally, loss of dendritic complexity in D1-MSNs facilitates CSDS susceptible behavior, and this morphological change in D1-MSNs results in decreased excitatory input to these neurons (32, 40, 41). These data demonstrate dynamic roles of MSN subtypes in stress outcomes with inhibition and reduced excitatory input in D1-MSN driving stress susceptible behavior. In contrast, repeated D2-MSN activation facilitates behavioral impairments induced by chronic stress. These findings corroborate clinical trials, which demonstrate that the D2R-agonist exerts antidepressant effects and a higher D2R sensitivity after antidepressant treatment (42). Considering that D2R has an inhibitory effect on neurons through Gi signaling, activating (e.g., using an agonist) or increasing the sensitivity (e.g., using antidepressants) of this receptor in the NAc may produce a pro-susceptibility through decreasing excitatory tone onto D2-MSNs (21, 42).

Considering that the results in this study extend beyond the previous literature on BDNF effects on reward circuitry in stress outcomes, it is important to highlight and discuss some important details. When overexpressing TrkB.t1 in D1-MSNs, we were able to induce a susceptible outcome in SSDS. We observed enhanced time in the corner zone, in mice expressing TrkB.t1 in D1-MSNS, after CSDS when compared with no-CSDS eYFP control conditions. However, there was no observed difference between CSDS-eYFP and CSDS-TrkB.t1. It is plausible that CSDS exerts a ceiling effect of stress susceptible behavior that blockade of BDNF signaling onto D1-MSNs, during CSDS, could not overcome. Interestingly, only SSDS in the D1-Cre-TrkB.t1 condition was able to trigger anhedonia. Unexpectedly, even the CSDS-eYFP mice did not show this behavior, although it is well-known that chronic stress is able to induce anhedonia (6, 21, 43, 44). This discrepancy in the results can be explained by the variability in the sucrose preference test across populations. For example, some studies report no alterations in sucrose preference during or after exposure to CSDS (45–47), and the same was observed for saccharin preference (48–51), demonstrating variation across labs and experiments. Thus, we chose to perform a battery of behavioral analyses, aiming to show a possible generalized stress susceptible behavior.

BDNF-TrkB signaling in the central nervous system has a wide range of functions, such as cellular survival, axon and dendrite growth, synaptogenesis, synaptic transmission, and synapse remodeling, which are essentially pro-plasticity and pro-neuronal activation (1, 52). Considering this, the overexpression of TrkB.t1 and blockade of BDNF-TrkB signaling, we are potentially blocking the plasticity and neuronal activation occurring through this signaling pathway. Our previous work indicates that D2-MSNs display enhanced dendritic spines and enhanced excitatory inputs in male mice that are susceptible to CSDS (21, 39). Our current results showed blockade of BDNF through overexpression of TkB.t1 in NAc D2-MSNs. This may prevent the plasticity effects occurring in D2-MSNs in stress susceptible conditions, thus resulting in a stress resilient response. Combined with previous studies, our results implicate that the D2-MSNs pathway is the mediator of BDNF-induced stress susceptibility likely through enhancing D2-MSN plasticity and response to excitatory inputs. Our data are consistent with D2-MSNs displaying enrichment of the TrkB receptor, compared with D1-MSNs (53, 54), and thus, it is not surprising that these BDNF-induced stress susceptible outcomes occur through this neuron subtype. In contrast, we showed that overexpression of TrkB.t1 and potential blockade of BDNF plasticity in NAc D1-MSNs induce a susceptible phenotype after an SSDS protocol, which is in line with reduced dendritic complexity and excitatory plasticity in these neurons in stress susceptible conditions (17, 21, 32, 41). Along with these previous studies, our results suggest that BDNF on D1-MSNs could drive a stress resilient response by preventing dendritic atrophy and reduced excitatory drive onto these neurons. While the effects of BDNF are likely enhanced on D2-MSNs, given the enriched expression of TrkB in these neurons, identification of molecules that create pro-plasticity effects on D1-MSNs could generate stress resilient outcomes. Indeed our previous work shows that blockade of the RhoA pathway, using inhibitors of RhoA or its downstream effecter Rho-kinase (ROCK), enhanced dendritic complexity or dendritic spines in D1-MSNs (32, 41) to cause a stress resilient outcome.

In conclusion, our results and previous findings implicate that D2-MSNs mediate stress susceptibility, through BDNF-TrkB signaling that may activate enhanced plasticity in these neuron subtypes. In contrast, BDNF-TrkB signaling in D1-MSNs has a protective role against social stress. Our studies are uncovering enhanced and detailed mechanisms in MSN subtypes in stress outcomes which can be informative for understanding mechanisms in stress precipitated clinical disorders.

## Data Availability Statement

The original contributions presented in the study are included in the article/Supplementary Material, further inquiries can be directed to the corresponding author.

## Ethics Statement

This animal study was reviewed and approved by Institutional Animal Care and Use Committee at the University of Maryland School of Medicine.

## Author Contributions

MP and ML: designed the study and drafted the manuscript. MP, DF, SC, GM-S, and RC: data acquisition and analyzed the data. MP, RC, MF, SI, CS, and ML: interpreted the data. All authors: approved the final version.

## Conflict of Interest

The authors declare that the research was conducted in the absence of any commercial or financial relationships that could be construed as a potential conflict of interest.

## Publisher’s Note

All claims expressed in this article are solely those of the authors and do not necessarily represent those of their affiliated organizations, or those of the publisher, the editors and the reviewers. Any product that may be evaluated in this article, or claim that may be made by its manufacturer, is not guaranteed or endorsed by the publisher.
