# Supplementary material for: The BDNF-TrkB Pathway Acts Through Nucleus Accumbens D2 Expressing Neurons to Mediate Stress Susceptible Outcomes
Source: Front Psychiatry. 2022 Jun 2;13:854494. doi: 10.3389/fpsyt.2022.854494 (PMC9200970; doi:10.3389/fpsyt.2022.854494)
Supplement: Supplementary file 1 [file Data_Sheet_1.DOCX]

**Results description**

Blockade of BDNF-TrkB signaling in D2-MSNs causes resilient outcomes to social stress in both sexes

Figure 2A: no-CSDS-eYFP: 82.52 ± 8.615s; no-CSDS-TrkB.t1: 78.16 ± 6.067s; CSDS-eYFP: 41.67 ± 4.794s; CSDS-TrkB.t1: 38.36 ± 12.87s).

Figure 2B: no-CSDS-eYFP: 17.41 ± 3.768s; no-CSDS-TrkB.t1: 15.15 ± 3.451s; CSDS-eYFP: 33.41 ± 7.541s; CSDS-TrkB.t1: 43.12 ± 12.00s

Figure 2C: Two-way ANOVA did not show significant differences in any factors and the *post hoc* Tukey's multiple comparisons test did not reveal statistical differences between groups (no-CSDS-eYFP: 141.5 ± 7.816s; no-CSDS-TrkB.t1: 150.7 ± 11.28s; CSDS-eYFP: 132.8 ± 13.70s; CSDS-TrkB.t1: 43.12 ± 12.00s)

Figure 2D: Two-way ANOVA did not show significant differences between groups (F_1,26_ = 0.567; *p* = 0.458) and the *post hoc* Tukey's multiple comparisons test did not reveal statistical differences between groups (no-CSDS-eYFP: 80.24 ± 3.317ml; no-CSDS-TrkB.t1: 79.54 ± 8.333ml; CSDS-eYFP: 82.03 ± 7.105ml; estresse-TrkB.t1: 87.42 ± 4.149ml).

Figure 2E: no-CSDS-eYFP: 148.0 ± 8.829s; no-CSDS-TrkB.t1: 139.9 ± 10.10s; CSDS-eYFP: 185.1 ± 9.747; CSDS-TrkB.t1: 183.8 ± 7.970s

Figure 3A: no-CSDS-eYFP: 58.49 ± 3.335s; no-CSDS-TrkB.t1: 66.52 ± 7.192s; CSDS-eYFP: 33.80 ± 4.763s; CSDS-TrkB.t1: 55.15 ± 8.586s

Figure 3B: no-CSDS-eYFP: 16.77 ± 1.417s; no-CSDS-TrkB.t1: 21.74 ± 5.064s; CSDS-eYFP: 44.09 ± 7.621s; CSDS-TrkB.t1: 22.63 ± 4.651s

Figure 3C: Two-way ANOVA did not show significant differences in any factors and the *post hoc* Tukey's multiple comparisons test did not reveal statistical differences between groups (no-CSDS-eYFP: 167.0 ± 15.28s; no-CSDS-TrkB.t1: 161.6 ± 5.685s; CSDS-eYFP: 149.0 ± 6.143s; CSDS-TrkB.t1: 170.2 ± 3.133s)

Figure 3D: no-CSDS-eYFP: 90.13 ± 2.253ml; no-CSDS-TrkB.t1: 89.27 ± 2.416ml; CSDS-eYFP: 62.11 ± 9.411ml; CSDS-TrkB.t1: 87.34 ± 2.551ml

Figure 3E: no-CSDS-eYFP: 144.1 ± 4.999s; no-CSDS-TrkB.t1: 135.9 ± 9.149s; CSDS-eYFP: 199.0 ± 10.58s; CSDS-TrkB.t1: 132.2 ± 14.48s

Figure 4A: no-CWDS-eYFP: no-target = 120.1 ± 10.66 target = 162.3 ± 13.22; no-CWDS-TrkB.t1: no-target = 80.94 ± 25.71 target = 159.5 ± 11.33; CWDS-eYFP: no-target = 132.3 ± 8.56 target = 158.6 ± 10.39; CWDS-TrkB.t1: no-target = 126.8 ± 8.56 target = 183.0 ± 12.90).

Figure 4B: Repeated measures two-way ANOVA showed significant differences only considering session (no-target x target) factor (F1,27 = 12.05; p ≤ 0.001). Sidak’s multiple comparisons test revealed statistical differences between no-target and target session only for TrkB.t1-no-CWDS group (p ≤ 0.01) (no-CWDS-eYFP no-target = 114.4 ± 8.46 target = 85.15 ± 12.61; no-CWDS-TrkB.t1: no-target = 159.7 ± 41.42 target = 81.28 ± 8.34; CWDS-eYFP: no-target = 112.9 ± 7.77 target = 98.33 ± 10.32; CWDS-TrkB.t1: no-target = 112.0 ± 6.78 target = 78.45 ± 9.62.

Figure 4C: Two-way ANOVA did not show significant differences in any factors for sucrose splash test, and the *post hoc* Tukey's multiple comparisons test did not reveal statistical differences between groups (no-CWDS-eYFP: 134.0 ± 11.87s; no-CWDS-TrkB.t1: 124.3 ± 24.86s; CWDS-eYFP: 102.2 ± 16.38s; CwDS-TrkB.t1: 104.3 ± 12.90s).

Figure 4D: Two-way ANOVA did not show significant differences in any factors for sucrose preference test, and the *post hoc* Tukey's multiple comparisons test did not reveal statistical differences between groups (no-CWDS-eYFP: 87.21 ± 3.21ml; no-CWDS-TrkB.t1: 69.20 ± 2.789ml; CWDS-eYFP: 83.15 ± 2.656ml; CWDS-TrkB.t1: 68.22 ± 7.28ml).

Figure 4E: Two-way ANOVA did not show significant differences in any factors for forced swim test, and the *post hoc* Tukey's multiple comparisons test did not reveal statistical differences between groups (no-CWDS-eYFP: 201.1 ± 4.16s; no-CWDS-TrkB.t1: 225.7 ± 15.97s; CWDS-eYFP: 244.9 ± 13.32s; CWDS-TrkB.t1: 231.1 ± 11.04s).

Blockade of BDNF-TrkB signaling in D1-MSNs induces a susceptible outcome after a subthreshold social defeat stress (SSDS)

Figure 5B: SSDS-eYFP: 59.20 ± 3.859s; SSDS-TrkB.t1: 45.14 ± 6.433s

Figure 5C: SSDS-eYFP: 21.60 ± 4.648s; SSDS-TrkB.t1: 47.45 ± 8.339s

Figure 5D: Unpaired T test did not show significant differences between groups (*p* = 0.4779; t = 0.7247 df = 18; SSDS-eYFP: 143.2 ± 7.313s; SSDS-TrkB.t1: 135.8 ± 7.068s)

Figure 5E: SSDS-eYFP: 77.17 ± 7.966; SSDS-TrkB.t1: 47.06 ± 10.38

Figure 5F: SSDS-eYFP: 142.5 ± 12.86s; SSDS-TrkB.t1: 21.9 ± 7.912

Blockade of BDNF-TrkB signaling in D2-MSNs prevents the intra-NAc BDNF induced susceptibility

**Figure 6B**: SSDS-Veh-eYFP: no-target 51.65 ± 8.346s; target 54.21 ± 8.965s; SSDS-Veh-TrkB.t1: no-target 49.46 ± 4.195s; target 66.06 ± 6.159s; SSDS-BDNF-eYFP: no-target 60.32 ± 7.087s; target 54.39 ± 12.51s; SSDS-BDNF-TrkB.t1: no-target 47.25 ± 3.403s; target 68.52 ± 5.233s.

**Figure 6C:** SSDS-Veh-eYFP: no-target 26.75 ± 4.838s; target 36.11 ± 9.113s; SSDS-Veh-TrkB.t1: no-target 19.76 ± 3.284s; target 29.60 ± 8.952s; SSDS-BDNF-eYFP: no-target 13.90 ± 1.841s; target 31.42 ± 5.076s; SSDS-BDNF-TrkB.t1: no-target 16.40 ± 3.792s; target 21.86 ± 5.856s

**Figure 6D:** SSDS-Veh-eYFP: 1.143 ± 0.03001; SSDS-Veh-TrkB.t1: 1.361 ± 0.1291; SSDS-BDNF-eYFP: 0.7973 ± 0.1578; SSDS-BDNF-TrkB.t1: 1.494 ± 0.1883

**Figure 6E**: SSDS-Veh-eYFP: 1.138 ± 0.1489; SSDS-Veh-TrkB.t1: 1.262 ± 0.3062; SSDS-BDNF-eYFP: 2.386 ± 0.4214; SSDS-BDNF-TrkB.t1: 1.306 ± 0.1957

**Supplemental figure**

Figure S1. Blocking BDNF-TrkB signaling in D1-MSNs induces a susceptible phenotype after one day (subthreshold) social defeat stress (SSDS) in male mice. Total time in the interaction zone during the target session when comparing figure 2 (CSDS) and figure 5 (SSDS) experiments. Both TrkB.t1 stressed groups (SSDS and CSDS) display social avoidance (p ≤ 0.01) when compared to no-CSDS. In contrast, only eYFP-CSDS (p ≤ 0.01), and no eYFP-SSDS, demonstrates social avoidance when compared to no-CSDS (N = 6-9; **p ≤ 0.01). Two-way ANOVA showed significant differences in the stress factor (F_2,42_ = 15.05; p ≤ 0.001). Post hoc Tukey's multiple comparisons test revealed statistical differences between no-SDS-eYFP *vs.* CSDS-eYFP (p ≤ 0.01), and between no-SDS-TrkB.t1-no-SDS and both stressed groups (p ≤ 0.01).
